# Carbon monoxide prevents TNF-α-induced eNOS downregulation by inhibiting NF-κB-responsive miR-155-5p biogenesis

**DOI:** 10.1038/emm.2017.193

**Published:** 2017-11-24

**Authors:** Seunghwan Choi, Joohwan Kim, Ji-Hee Kim, Dong-Keon Lee, Wonjin Park, Minsik Park, Suji Kim, Jong Yun Hwang, Moo-Ho Won, Yoon Kyung Choi, Sungwoo Ryoo, Kwon-Soo Ha, Young-Guen Kwon, Young-Myeong Kim

**Affiliations:** 1 Departments of Molecular and Cellular Biochemistry, Kangwon National University School of Medicine, Chuncheon, Gangwon-do, South Korea; 2 Departments of Obstetrics and Gynecology, Kangwon National University School of Medicine, Chuncheon, Gangwon-do, South Korea; 3 Department of Neurobiology, Kangwon National University School of Medicine, Chuncheon, Gangwon-do, South Korea; 4 Department of Integrative Bioscience and Biotechnology, Konkuk University, Seoul, South Korea; 5 Department of Biology, College of Natural Sciences, Kangwon National University, Chuncheon, Gangwon-do, South Korea; 6 Department of Biochemistry, College of Life Science and Biotechnology, Yonsei University, Seoul, South Korea

## Abstract

Heme oxygenase-1-derived carbon monoxide prevents inflammatory vascular disorders. To date, there is no clear evidence that HO-1/CO prevents endothelial dysfunction associated with the downregulation of endothelial NO synthesis in human endothelial cells stimulated with TNF-α. Here, we found that the CO-releasing compound CORM-2 prevented TNF-α-mediated decreases in eNOS expression and NO/cGMP production, without affecting eNOS promoter activity, by maintaining the functional activity of the *eNOS* mRNA 3′-untranslated region. By contrast, CORM-2 inhibited MIR155HG expression and miR-155-5p biogenesis in TNF-α-stimulated endothelial cells, resulting in recovery of the 3′-UTR activity of *eNOS* mRNA, a target of miR-155-5p. The beneficial effect of CORM-2 was blocked by an NF-κB inhibitor, a miR-155-5p mimic, a HO-1 inhibitor and siRNA against HO-1, indicating that CO rescues TNF-α-induced eNOS downregulation through NF-κB-responsive miR-155-5p expression via HO-1 induction; similar protective effects of ectopic HO-1 expression and bilirubin were observed in endothelial cells treated with TNF-α. Moreover, heme degradation products, except iron and *N*-acetylcysteine prevented H_2_O_2_-mediated miR-155-5p biogenesis and eNOS downregulation. These data demonstrate that CO prevents TNF-α-mediated eNOS downregulation by inhibiting redox-sensitive miR-155-5p biogenesis through a positive forward circuit between CO and HO-1 induction. This circuit may play an important preventive role in inflammatory endothelial dysfunction associated with human vascular diseases.

## Introduction

Nitric oxide (NO) produced from L-arginine by endothelial nitric oxide synthase (eNOS) exerts multiple beneficial functions, such as vasorelaxation, anti-inflammation and anti-apoptosis, in the vasculature and thus plays a critical role in vascular homeostasis and disorders.^1^ Abnormally decreased eNOS expression and activity, leading to impairment of the NO/cGMP pathway, are considered major contributors to the pathogenesis of various human diseases associated with endothelial dysfunction, such as hypertension, atherosclerosis and preeclampsia.^1, 2, 3^

A great deal of evidence shows that the eNOS/NO pathway can be regulated in response to a wide variety of pathophysiological stimuli.^4, 5, 6, 7, 8, 9, 10^ The activity of eNOS is mostly regulated by multiple post-translational processes, such as Ca^2+^-dependent dimerization, phosphorylation, subcellular localization and protein–protein interactions.^4, 5^ Although eNOS is known as a constitutive enzyme, its expression can be modulated at both transcriptional and post-transcriptional levels by various pathophysiological stresses and stimuli.^6, 7, 8, 9, 10^ Indeed, shear stress, estrogen and statin elicit a transcriptional increase in eNOS expression,^6, 7, 8^ whereas inflammatory nuclear factor-κB (NF-κB) activators, including lipopolysaccharide (LPS), tumor necrosis factor (TNF)-α, oxidized low-density lipoprotein (oxLDL) and reactive oxygen species (ROS) including H_2_O_2_, suppress eNOS expression by decreasing *eNOS* mRNA stability.^9, 10, 11^ Thus, eNOS-derived NO production is regulated via multiple distinct mechanisms.

A recent study demonstrated that eNOS expression is negatively regulated at the post-transcriptional level by upregulating microRNA (miR)-155-5p via NF-κB, which is activated by a number of inflammatory stimuli including TNF-α.^9^ NF-κB-responsive miR-155-5p directly binds to the 3′-untranslated region (3′-UTR) of *eNOS* mRNA, resulting in decreased miRNA degradation and expression. This evidence indicates that the pathogenesis of vascular diseases, including atherosclerosis, obese/type 2 diabetic vascular complications and preeclampsia, can be associated with endothelial dysfunction by decreasing eNOS expression via NF-κB activation.^2, 3, 10, 12^ Therefore, NF-κB plays an important role in inflammation-associated endothelial dysfunction and vascular disorders.

Heme oxygenase (HO) catalyzes heme degradation to produce biliverdin (which is ultimately converted to bilirubin), iron and carbon monoxide (CO).^13^ Among these products, CO plays the most important role in preventing vascular dysfunction and maintaining vascular physiology or homeostasis^13^ in both eNOS-dependent and independent manners.^14, 15^ HO-1-derived CO also acts as a potent NF-κB inhibitor to prevent TNF-α-induced vascular inflammation in endothelial cells.^16^ Interestingly, HO-1 overexpression can improve vascular function by restoring decreased eNOS levels after exposure to TNF-α and oxLDL;^17^ however, the mechanism by which HO-1/CO prevents eNOS downregulation under inflammatory conditions has been largely unexplored.

Here, we found that HO-1-derived CO restores TNF-α-induced eNOS downregulation by inhibiting NF-κB-responsive miR-155-5p expression in endothelial cells. These results provide a new mechanistic explanation for the beneficial effect of HO-1/CO on eNOS restoration and vascular homeostasis coupled with inflammatory vascular disease.

## Materials and methods

### Materials

Cell culture media and supplements, Lipofectamine RNAiMAX and Lipofectamine 3000 were purchased from Invitrogen Life Technologies (Carlsbad, CA, USA). 4-Amino-5-methylamino-2,7-difluorofluorescein (DAF-FM) diacetate and *N*^5^-(1-iminoethyl)-L-ornithine HCl (L-NIO) were obtained from Molecular Probes (Eugene, OR, USA) and Alexis Biochemicals (San Diego, CA, USA), respectively. Antibodies against human eNOS and HO-1 were purchased from BD Biosciences (San Jose, CA, USA). Other antibodies were purchased from Santa Cruz Biotechnology (Santa Cruz, CA, USA). miRNAs, siRNA and miRNA assay chemicals were purchased from QIAGEN (Hilden, Germany). Luciferase reporter assay kits were obtained from Promega (Madison, WI, USA). The TNF-α and a cGMP assay kits were purchased from R&D Systems (Minneapolis, MN, USA). Oxyhemoglobin (oxyHb) was prepared by reduction of human hemoglobin (Sigma-Aldrich, St. Louis, MO, USA) with a 20-fold excess amount of sodium dithionite for 20 min, followed by gel filtration using a pre-packed disposable column (PD-10, Pharmacia, Uppsala, Sweden) that had been pre-equilibrated with 50 mM Tris-HCl (pH 7.4). Sn(IV) protoporphyrin IX dichloride (SnPP) and biliverdin were obtained from Frontier Scientific (Logan, UT, USA). RuCl_3_, CORM-2, hemin, bilirubin and iron chloride (FeCl_2_) were purchased from Sigma-Aldrich.

### HUVEC culture and treatment

HUVECs were cultured as described previously,^9^ and only passages 3–6 were used. In brief, cells were grown in M199 medium supplemented with 20% fetal bovine serum, 100 U ml^−1^ penicillin, 100 ng ml^−1^ streptomycin, 3 ng ml^−1^ basic fibroblast growth factor and 5 U/ml heparin at 37 °C in a humidified CO_2_ incubator. CORM-2 and RuCl_3_ were dissolved in DMSO to generate a 100 mM stock solution. HUVECs were pretreated for 3 h with the indicated concentrations, 200 μM CORM-2 or 200 μM RuCl_3_ (as a control), followed by stimulation with TNF-α (10 ng ml^−1^) for 16 h.

### Transfection with miRNAs, siRNA and pcHO-1

HUVECs were seeded into six-well plates coated with 2% gelatin at a density of 2 × 10^5^ cells per well and maintained for 1 day. The cells were cultured in serum-free medium for 2 h and then transfected with antagomiR-155-5p (80 nM), miR-155-5p mimic (80 nM), control miRNA (80 nM), HO-1 siRNA (100 nM), scrambled control (100 nM), pcDNA3.0 (1 μg ml^−1^) or pcHO-1 (1 μg ml^−1^, provided by Dr Jozef Dulak, Jagiellonian University, Czech Republic) in Opti-MEM-reduced serum medium using Lipofectamine RNAiMAX for miRNA and siRNA or Lipofectamine 3000 for plasmid DNA, according to the manufacturer′s instructions. After incubation for 4 h, the cells were further recovered in fresh medium for 24 h and used to assay the expression levels of the target genes.

### Reporter gene assay

HUVECs were transfected with 1 μg ml^−1^ of pGL3-eNOS promoter (1.6 kb)-Luc construct (containing the 1.6-kb region), pGL3-MIR155HG promoter (2.0 kb)-Luc construct, pGL3-NF-κB promoter, psiCHECK-2-*eNOS* 3′-UTR-wild/mutant reporter constructs or each basic plasmid using Lipofectamine 3000.^9^ After a 4-h incubation, the cells were recovered in fresh medium for 24 h. Cells were pre-incubated with CORM-2 (200 μM) for 3 h and stimulated with TNF-α (10 ng ml^−1^) for 16 h. Reporter gene activity was assayed using a luciferase assay system or a dual-luciferase report assay kit.

### NO, ROS and cGMP measurements

Cells were transfected with miR-155-5p mimic or antagomiR-155-5p for 24 h, followed by stimulation with TNF-α (10 ng ml^−1^) for 16 h following pretreatment with RuCl_3_ (200 μM) or CORM-2 (200 μM) for 3 h. Cells were incubated with DAF-FM diacetate (5 μM) for 30 min, and intracellular NO levels were determined using a confocal laser microscope.^9^ Intracellular ROS production in HUVECs was measured by confocal microscopy using the fluorescent ROS indicator dye DCFH_2_-DA.^18^ For cGMP measurement, HUVECs were treated with TNF-α alone or in combination with RuCl_3_ or CORM-2 for 16 h as described above and further incubated with the phosphodiesterase inhibitor 3-isobutyl-1-methylxanthine (IBMX, 500 μM) or the HO inhibitor SnPP (25 μM) for 6 h. In another set of experiment, HUVECs were treated with TNF-α alone or in combination with CORM-2 for 16 h, harvested and washed with fresh medium to remove the TNF-α and CORM-2. The cells were replated at a density of 5 × 10^4^ cells per 35 mm well (in a six-well plate) or co-cultured with human vascular smooth muscle cells at a ratio of 1 to 3 in the presence of IBMX and SnPP for 12 h. Intracellular cGMP levels were determined with an ELISA kit.

### PCR analysis

Total miRNAs were isolated from HUVECs using the miRNeasy Mini kit. cDNAs were prepared from 1 μg of miRNAs using a miScript II RT kit. Quantitative real-time PCR (qRT-PCR) was performed with the miScript SYBR Green PCR Kit according to the manufacturer’s instructions. Levels of miR-155-5p were analyzed using the miScript Primer Assay with miR-155-5p-specific and universal primers and normalized to SNORD-95. In addition, eNOS and MIR155HG mRNA levels were quantitated by qRT-PCR with target-specific primers using cDNAs synthesized from total mRNAs as described previously.^9^

### Western blot analysis

HUVECs were suspended in RIPA buffer (50 mM Tris-HCl, pH 8.0, 150 mM NaCl, 1% Nonidet P-40, 0.5% deoxycholic acid, 0.1% sodium dodecyl sulfate (SDS)) and incubated on ice for 30 min for complete cell lysis. Cell debris was removed by centrifugation at 12 000 × *g* for 15 min. Lysates (30 μg of protein) were separated by SDS-polyacrylamide gel electrophoresis and target protein levels were determined by Western blot analysis.^9^

### ChIP analysis

HUVECs were treated with TNF-α (10 ng ml^−1^) for 12 h after pretreatment with RuCl_3_ (200 μM) or CORM-2 (200 μM) for 3 h. DNA/protein crosslinking was performed by incubating the cells for 20 min at 37 °C in 1% formaldehyde. After sonication, chromatin was immunoprecipitated overnight with 1 μg of anti-NF-κB 65 antibody (sc-372, Santa Cruz Biotechnol.). Targeted promoter sequences of MIR155HG were amplified by PCR using primer pairs spanning MIR155HG-specific promoter regions containing the NF-κB binding site at –1150.^9^ The products (~183 bp) were identified on a 2% agarose gel.

### Preparation of cytosolic and nuclear fractions

HUVECs were treated with RuCl_3_ (200 μM) or CORM-2 (200 μM) for 3 h, followed by treatment with TNF-α (10 ngml^−1^) for 2 h. After washing twice with phosphate-buffered saline. The cells were scraped into buffer A (10 mM HEPES, pH 7.9, 0.1 mM EDTA, 10 mM KCl, 0.1 mM EGTA) and centrifuged at 14 000 *g* for 10 min at 4 °C. The cell pellets were suspended in buffer A plus 0.1% Nonidet P-40. After centrifugation at 12 000 *g* for 5 min, the nuclear pellets were suspended in 20 mM HEPES (pH 7.9) containing 0.4 M NaCl, 1 mM EDTA and 1 mM EGTA and lysed by three cycles of freezing and thawing. After incubation on ice for 30 min, the nuclear lysates were centrifuged at 12 000 *g* for 10 min. The supernatant was analyzed by Western blotting.

### Nuclear translocation of NF-κB p65

HUVECs were treated with TNF-α (10 ng ml^−1^) for 2 h after pretreatment with RuCl_3_ (200 μM) or CORM-2 (200 μM) for 3 h and fixed in 3.7% formaldehyde for 15 min at room temperature. After washing gently, the cells were permeabilized with 0.1% saponin and incubated with an antibody (1:100) against the NF-κB p65 subunit for 2 h, followed by incubation with anti-goat IgG-TRITC (1:200) for 1 h. For nuclear staining, the cells were further incubated with DAPI (1 μg ml^−1^, Sigma-Aldrich) for 30 min. After mounting, nuclear translocation of NF-κB p65 was observed using a confocal microscope.

### Statistical analysis

Quantitative data are expressed as the mean±s.d. from three independent experiments performed in triplicate. All statistical analyses were performed with GraphPad Prism 5.0 for windows (GraphPad Software, San Diego, CA, USA). Statistical significance was determined using one-way analysis of variance or the unpaired Student’s *t* test, depending on the number of experimental groups analyzed. Significance was established at a *P* value <0.05.

## Results

### CORM-2 restores TNF-α-mediated suppression of eNOS expression

**Figure 1:**
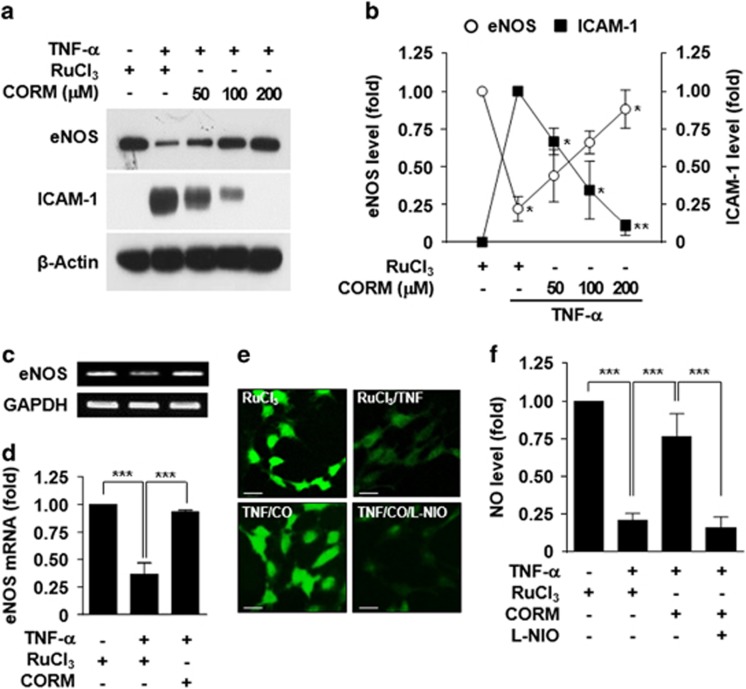
CORM-2 prevents tumor necrosis factor (TNF)-α-mediated eNOS downregulation. (**a**–**d**) HUVECs were pretreated with the indicated concentrations or 200 μM of CORM-2 or RuCl_3_ for 3 h and stimulated with TNF-α (10 ng ml^−1^) for 16 h. (**a**, **b**) eNOS and ICAM-1 protein levels were determined by western blotting, and protein band intensities were measured by ImageJ. (**c**, **d**) eNOS mRNA levels were measured by RT-PCR (upper) and qRT-PCR (lower). (**e**, **f**) Cells pretreated with CORM-2 or RuCl_3_ for 3 h or L-NIO (1 mM) for 1 h were stimulated with TNF-α for 16 h. Intracellular NO levels were determined by confocal microscopy using DAF-FM diacetate, and the fluorescence density was quantitated by ImageJ. Scale bars: 10 μM. *n*=3. *P<0.05, **P<0.01 and ***P<0.001.

TNF-α, which is a risk factor for various inflammatory cardiovascular diseases, such as atherosclerosis, diabetic vascular complication and preeclampsia, causes endothelial dysfunction by suppressing eNOS expression.^19, 20^ By contrast, CO inhibits TNF-α-mediated inflammation and vascular dysfunction.^21, 22^ We examined whether CO regulates the eNOS/NO pathway in TNF-α-treated HUVECs. Treatment with TNF-α decreased eNOS protein levels and increased intercellular adhesion molecule 1 (ICAM-1, an indicator of vascular inflammation) expression in HUVECs and both effects were blocked in a dose-dependent manner by treatment with CORM-2 (a CO-releasing molecule), compared with the negative control RuCl_3_ (Figure 1a and b). Consistent with these data, TNF-α decreased *eNOS* mRNA, which was effectively blocked by treatment with CORM-2 but not with RuCl_3_ (Figure 1c and d). As expected, the TNF-α-mediated decrease in NO production was effectively recovered by CORM-2 treatment, and this recovery effect was abrogated by the eNOS inhibitor L-NIO (Figure 1e and f), indicating that CO rescues NO production in an eNOS-dependent manner. These results suggest that CO prevents the TNF-α-induced decrease in eNOS expression and NO production.

### CO restores TNF-α-induced eNOS downregulation by inhibiting miR-155-5p biogenesis

**Figure 2:**
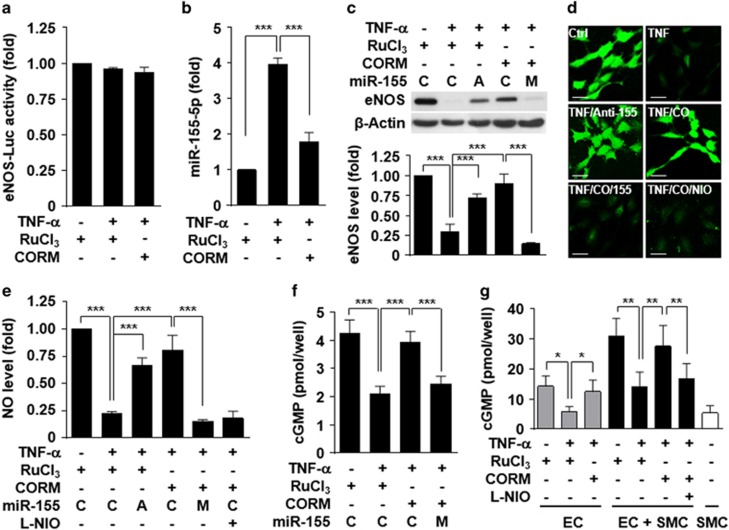
CORM-2 restores TNF-α-induced eNOS downregulation by inhibiting miR-155-5p biogenesis. HUVECs were transfected with an eNOS promoter-Luc construct, control miRNA (C) miR-155-5p mimic (M) or antagomiR-155-5p (A) and stimulated with TNF-α in the presence of RuCl_3_ (200 μM) or CORM-2 (200 μM) for 16 h. (**a**) Luciferase activity was determined in cell lysates. (**b**, **c**) Levels of miR-155-5p and eNOS protein were determined by qRT-PCR and western blotting, respectively. (**d**, **e**) Intracellular NO levels were determined by confocal microscopy using DAF-FM diacetate, and fluorescence intensity was quantitated by ImageJ. Scale bars: 10 μM. (**f**, **g**) HUVECs transfected with control miRNA or miR-155-5p mimic were stimulated with TNF-α in the presence of RuCl_3_ or CORM-2. (**f**) After washing, the cells were further incubated in fresh medium for 6 h, and intracellular cGMP levels were determined using an ELISA kit. (**g**) Pre-stimulated endothelial cells (ECs) were replated with or without smooth muscle cells (SMC) and cultured for 12 h. Intracellular cGMP levels were determined. *n*=3. *P<0.05, **P<0.01 and ***P<0.001.

To investigate whether CO transcriptionally regulates human eNOS expression, we examined the effect of CO on eNOS promoter activity in HUVECs. The promoter activity was not affected by TNF-α alone or in combination with CORM-2 (Figure 2a), suggesting that neither TNF-α nor CO regulates transcriptional eNOS expression. Because a recent study showed that eNOS expression is post-transcriptionally regulated by elevating TNF-α-induced miR-155-5p biogenesis, we next examined whether CO regulates miR-155-5p expression in HUVECs. TNF-α treatment resulted in an almost fourfold increase in miR-155-5p, which was effectively reduced by CORM-2 (Figure 2b). In addition, CORM-2 and antagomiR-155-5p rescued the TNF-α-mediated decreases in the eNOS level and NO production (Figure 2c and e). Interestingly, transfection with a miR-155-5p mimic abolished the recovery effect of CORM-2 on the TNF-α-mediated decrease in eNOS expression and NO production (Figure 2c–e). These results suggest that CO restores TNF-α-mediated attenuation of eNOS-specific NO production, as confirmed by using the eNOS inhibitor L-NIO (Figure 2d and e), by inhibiting miR-155-5p biogenesis. Moreover, CO prevented a TNF-α-induced decrease in cGMP production in HUVECs, and this effect was reversed by transfection with the miR-155-5p mimic (Figure 2f). We further examined the effect of CO and miR-155-5p on cGMP production in a co-culture model of endothelial and smooth muscle cells, which is a mimic vascular system (Figure 2g). Pretreatment of HUVECs with TNF-α significantly decreased cGMP production in the co-culture system with smooth muscle cells compared with untreated endothelial cells, and the detrimental effect of TNF-α was abolished by co-treatment with CORM-2 (Figure 2g).

### CO inhibits TNF-α-induced transcriptional biogenesis of miR-155-5p

**Figure 3:**
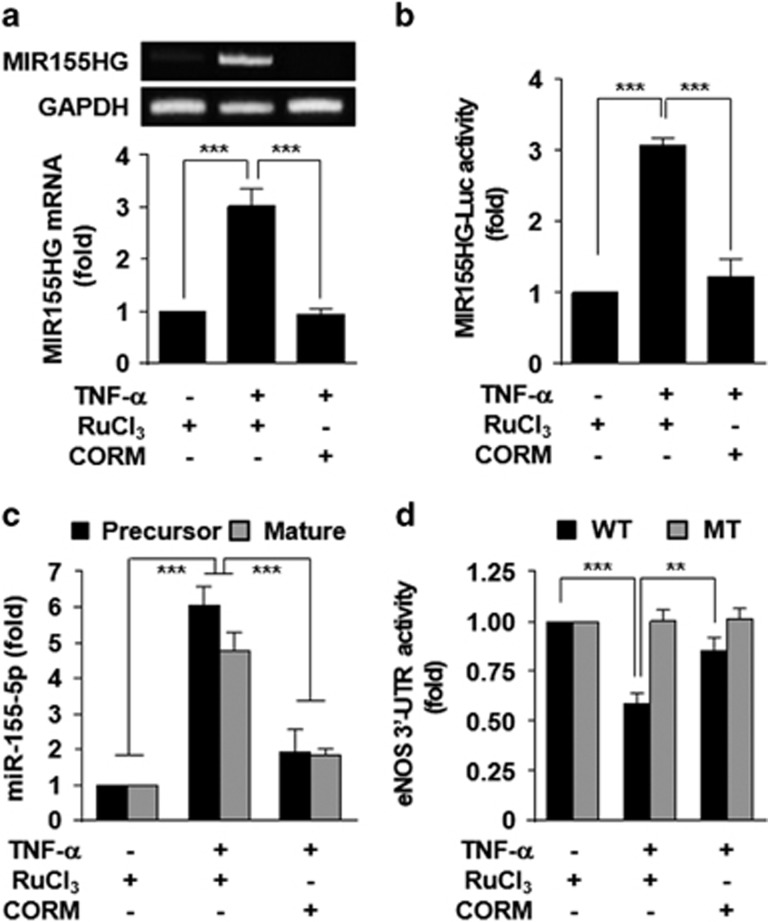
CORM-2 inhibits TNF-α-mediated biogenesis of miR-155-5p that targets the 3′-UTR of *eNOS*. HUVECs were transfected with or without MIR155HG promoter-Luc construct or psiCHECK-2-*eNOS* 3′-UTR reporter constructs (wild type, WT; mutant, MT) and stimulated with TNF-α in the presence of RuCl_3_ (200 μM) or CORM-2 (200 μM). (**a**) MIR155HG expression was determined by RT-PCR (upper) and qRT-PCR (lower). (**b**) MIR155HG promoter-Luc activity was determined in cell lysates. (**c**) Precursor and mature miR-155-5p levels were determined by qRT-PCR. (**d**) Luciferase activity of *eNOS* 3′-UTR was determined in cell lysates. *n*=3. **P<0.01; ***P<0.001.

Because TNF-α increased the miR-155-5p level (Figure 2b), we investigated whether CO regulates TNF-α-induced transcriptional expression of the miR-155 host gene (MIR155HG). As expected, TNF-α significantly increased *MIR155HG* mRNA expression and promoter activity in HUVECs, and these increases were abolished by CORM-2 (Figure 3a and b). Consequently, CORM-2 blocked the TNF-α-mediated increases in both miR-155-5p precursor and mature forms (Figure 3c). These results suggest that CO inhibits TNF-α-induced miR-155-5p biogenesis at the transcriptional level. Since miR-155-5p negatively regulates eNOS expression by targeting the 3′-UTR of its mRNA,^9^ we further examined whether CO would regulate the biological function of the *eNOS* 3′-UTR using the luciferase reporter gene assay. Treatment with TNF-α decreased wild-type *eNOS* 3′-UTR-based luciferase activity, but not the reporter activity of its mutant 3′-UTR that is created at a complementary binding site with miR-155-5p, and the decreased wild-type reporter activity was significantly recovered by CORM-2 treatment (Figure 3d). These results suggest that CO prevents TNF-α-mediated transcriptional biogenesis of miR-155-5p, which negatively regulates eNOS expression by targeting the 3′-UTR of *eNOS* mRNA.

### CO blocks TNF-α-induced miR-155-5p biogenesis via NF-κB inhibition

**Figure 4:**
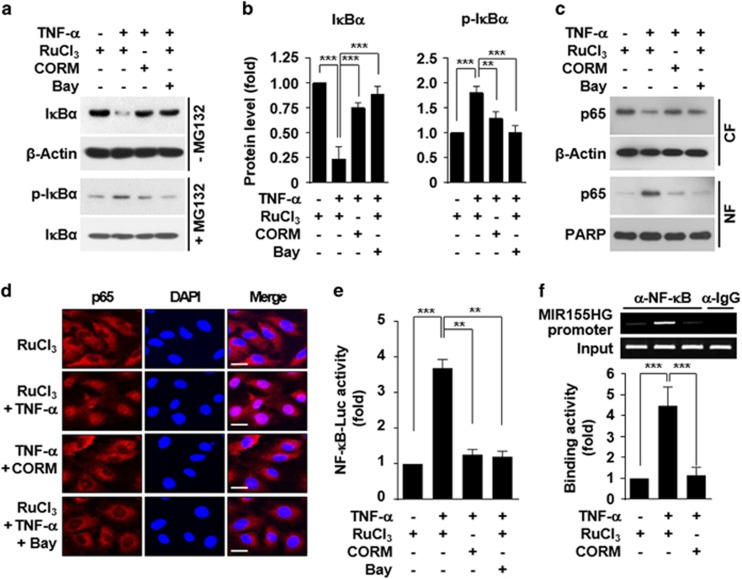
CORM-2 inhibits the TNF-α-induced NF-κB binding to the MIR155HG promoter. HUVECs were transfected with or without an NF-κB-Luc construct and stimulated with TNF-α in the presence or absence of RuCl_3_ (200 μM), CORM-2 (200 μM) or Bay 11-7082 (5 μM). The cells were stimulated with TNF-α. (**a**, **b**) After 30 min of stimulation, p-IκBα and IκBα levels were determined by western blotting and quantified by ImageJ. (**c**, **d**) After 2 h of stimulation, nuclear translocation of NF-κB p65 was determined by western blotting and confocal microscopy. Scale bars: 10 μm. (**e**, **f**) After 12 h of stimulation, luciferase activity was determined in cell lysates. ChIP analysis was also performed to evaluate the binding activity of NF-κB to the κB-binding site at −1150 of the MIR155HG promoter. *n*=3. **P<0.01, ***P<0.001.

Since TNF-α can induce miR-155-5p biogenesis via NF-κB activation in HUVECs,^9^ we next examined whether CO inhibits TNF-α-mediated NF-κB activation and MIR155HG promoter activity. As expected, TNF-α effectively promoted phosphorylation and degradation of inhibitor of κB (IκB), which were blocked by co-treatment with CORM-2 (Figure 4a and b). We next examined the effect of CO on TNF-α-mediated NF-κB nuclear translocation because cytosolic NF-κB liberated from IκB translocates to the nucleus, where it participates in the transcriptional activation of target genes.^23^ CORM-2 inhibited TNF-α-induced nuclear translocation of the NF-κB subunit p65, as determined by Western blot analysis and immunocytochemistry (Figure 4c and d). CORM-2 also blocked the TNF-α-mediated increase in NF-κB reporter activity (Figure 4e). In addition, chromatin immunoprecipitation analysis showed that CORM-2 inhibited TNF-α-mediated NF-κB binding to the MIR155HG promoter at the −1150 region (Figure 4f), and all the effects of CORM-2 were similar to those of the NF-κB inhibitor Bay 11-7082 (Figure 4a–f). These results suggest that CO suppresses transcriptional biogenesis of miR-155-5p in TNF-α-stimulated HUVECs by inhibiting IKK-dependent NF-κB activation.

### CO inhibits the TNF-α-mediated miR-155-5p/eNOS/NO axis via HO-1 induction

**Figure 5:**
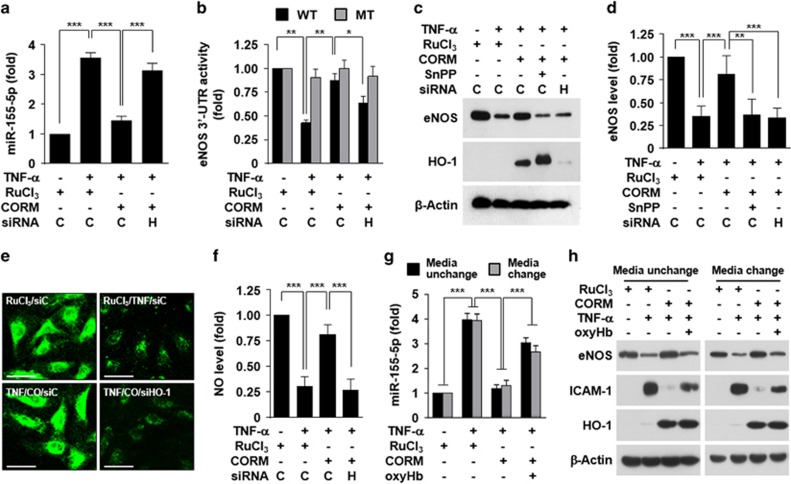
CO inhibits the TNF-α-induced miR-155-5p/eNOS pathway via HO-1 induction. HUVECs were transfected with scrambled control siRNA (C), HO-1 siRNA (H) or psiCHECK-2-*eNOS* 3′-UTR reporter constructs (wild type, WT; mutant, MT) and stimulated with TNF-α in the presence of RuCl_3_ (200 μM), CORM-2 (200 μM) or SnPP (20 μM). (**a**) miR-155-5p levels were determined by qRT-PCR. (**b**) Luciferase activity was determined in cell lysates. (**c**, **d**) eNOS and HO-1 protein levels were determined by western blotting and quantitated by ImageJ. (**e**, **f**) Intracellular NO levels were determined by confocal microscopy. Scale bars: 10 μm. (**g**, **h**) Cells were treated with RuCl_3_ or CORM-2 for 8 h, followed by stimulation in the same or fresh medium with TNF-α in the presence or absence of oxyHb (60 μM) for 16 h. miR-155-5p levels and eNOS and ICAM-1 protein levels were determined by qRT-PCR and western blotting. *n*=3. *P<0.05, **P<0.01 and ***P<0.001.

CO that is produced from heme degradation by HO-1 plays an important role in regulating endothelial cell function.^13, 24^ Thus, we examined whether the effect of exogenous CO on the miR-155-5p/eNOS pathway occurs in conjunction with the induction of HO-1. Transfection with an siRNA for HO-1 or treatment with the HO inhibitor SnPP reversed the suppressive effect of CORM-2 on miR-155-5p biogenesis in HUVECs treated with TNF-α (Figure 5a), subsequently leading to decreased CORM-2-mediated recovery of *eNOS* 3′-UTR reporter activity, but not its mutant activity (Figure 5b). Furthermore, CORM-2 treatment induced HO-1 expression in conjunction with eNOS restoration, which was abolished by treatment with SnPP and HO-1 siRNA, in TNF-α-stimulated HUVECs (Figure 5c and d). As a result, treatment with HO-1 siRNA effectively suppressed the recovery effect of CORM-2 on the TNF-α-mediated decrease in NO production (Figure 5e and f). These results suggest that exogenous CO inhibits the TNF-α-mediated increase in miR-155-5p biogenesis, resulting in restoration of eNOS expression via HO-1 induction. Next, we examined the effects of CORM-2-dependent HO-1 induction on TNF-α-induced eNOS downregulation. After endothelial cells were pretreated with COMR-2 for 8 h, some of the cells were continuously cultured in the same medium, while the others were washed and cultured in fresh medium without CORM-2, followed by stimulation with TNF-α alone or in combination with oxyHb. The inhibitory effects of CORM-2 on TNF-α-induced miR-155-5p expression, eNOS downregulation and NF-κB-responsive ICAM-1 expression were not significantly different in the two experimental conditions, and the inhibitory effects were similarly reversed by oxyHb in both groups (Figure 5g and h), suggesting that CORM-2-mediated HO-1 induction and subsequent endogenous CO production play an important role in regulating the TNF-α-mediated miR-155-5p/eNOS axis.

### Endogenous HO-1 inhibits the TNF-α-induced miR-155-5p/eNOS/NO axis

**Figure 6:**
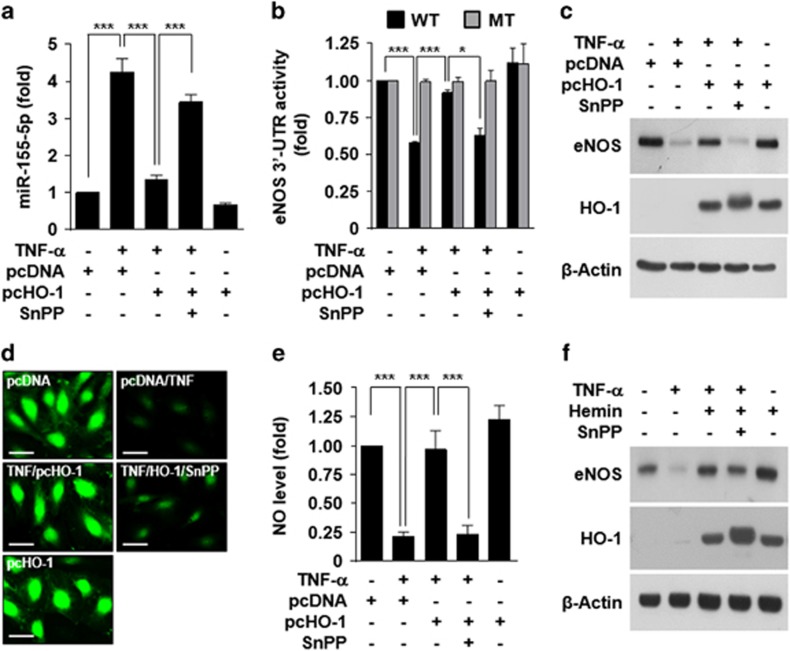
HO-1 overexpression inhibits the TNF-α-induced miR-155-5p/eNOS axis. (**a–e**) HUVECs were transfected with pcDNA3.0 or the pcHO-1 gene and stimulated with or without TNF-α alone or in combination with SnPP (20 μM). (**a**) miR-155-5p levels were determined by qRT-PCR. (**b**) The 3′-UTR activity was determined in lysates of cells transfected with psiCHECK-2-*eNOS* 3′-UTR constructs (wild type, WT; mutant, MT). (**c**) eNOS and HO-1 protein levels were determined by western blotting. (**d**, **e**) Intracellular NO levels were determined by confocal microscopy using DAF-FM diacetate. Scale bars: 10 μm. (**f**) Cells were treated with hemin (20 μM) alone or in combination with SnPP for 3 h, followed by stimulation with TNF-α in the presence or absence of SnPP. eNOS and HO-1 protein levels were determined by Western blotting. *n*=3. *P<0.05 and ***P<0.001.

We further examined the role of HO-1 activity in the miR-155-5p/eNOS/NO axis in endothelial cells. Ectopic expression of HO-1 inhibited TNF-α-induced miR-155-5p biogenesis, and this inhibition was recovered by SnPP (Figure 6a). In addition, HO-1 overexpression prevented the TNF-α-induced decrease in *eNOS* 3′-UTR activity, but not its mutant activity, and the preventive effect was reversed by SnPP (Figure 6b). Similar protective effects of endogenous HO-1 on eNOS expression and NO production were observed in HUVECs treated with TNF-α (Figure 6c–e). Moreover, treatment with hemin, a strong HO-1 inducer, restored the TNF-α-induced decrease in eNOS expression and this restoration was blocked by SnPP (Figure 6f). These results suggest that HO-1 activity plays an important role in preventing post-translational eNOS downregulation in HUVECs treated with TNF-α via inhibition of miR-155-5p biogenesis.

### CO and bilirubin rescues the TNF-α-induced eNOS downregulation

**Figure 7:**
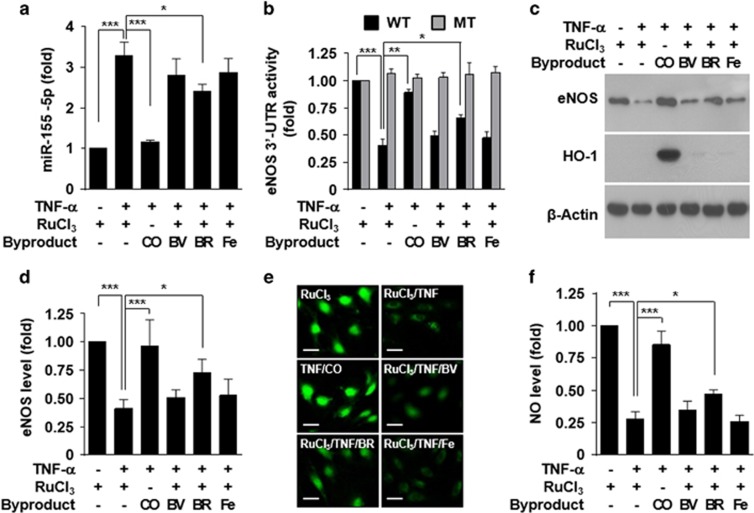
CO and bilirubin restore TNF-α-induced eNOS downregulation. HUVECs transfected with or without psiCHECK-2-*eNOS*-3′-UTR constructs (wild type, WT; mutant, MT) were pretreated with RuCl_3_ (200 μM), CORM-2 (CO, 200 μM), biliverdin (BV, 20 μM), bilirubin (BR, 20 μM) or FeCl_2_ (Fe, 20 μM), followed by stimulation with TNF-α. (**a**) miR-155-5p levels were determined by qRT-PCR. (**b**) Luciferase activity was determined in cell lysates. (**c**, **d**) eNOS and HO-1 levels were determined by western blotting. (**e**, **f)** Intracellular NO levels were determined by confocal microscopy. Scale bars: 10 μm. NS=no significant. *n*=3. *P<0.05, **P<0.01 and ***P<0.001.

HO-1 degrades heme to CO, iron and biliverdin, which is rapidly converted to bilirubin by biliverdin reductase.^13^ Thus, we examined which byproducts of heme play an important role in regulating the miR-155-5p/eNOS axis. Treatment with CO or bilirubin, but not with biliverdin or iron, inhibited miR-155-5p biogenesis in HUVECs treated with TNF-α (Figure 7a). Both CO and bilirubin showed recovery effects on *eNOS* 3′-UTR activity, eNOS expression and NO production in HUVECs stimulated with TNF-α (Figure 7b–f). However, CO had a stronger effect than bilirubin (Figure 7a–f) and stimulated HO-1 expression (Figure 7c). These results suggest that HO-1-derived CO and bilirubin restore eNOS downregulation in TNF-α-stimulated HUVECs by inhibiting miR-155-5p biogenesis.

### Heme degradation products, except iron, prevent redox-sensitive miR-155-5p biogenesis and eNOS downregulation

**Figure 8:**
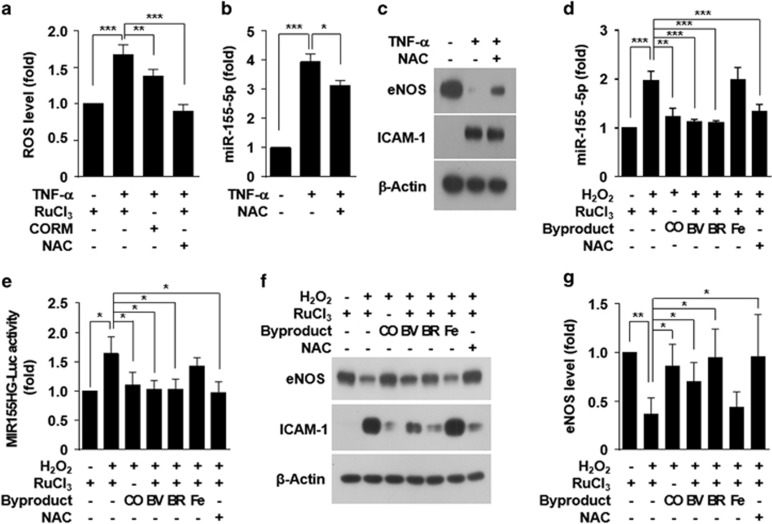
Heme degradation products, except iron, prevent H_2_O_2_-induced eNOS downregulation. HUVECs were treated RuCl_3_ (200 μM), CORM-2 (CO, 200 μM), biliverdin (BV, 20 μM), bilirubin (BR, 20 μM), FeCl_2_ (Fe, 20 μM) or NAC (1 mM) for 3 h, followed by treatment with TNF-α (10 ng ml^−1^) or H_2_O_2_ (200 μM) for 16 h. (**a**) Intracellular ROS levels were determined by confocal microscopy using DCFH_2_-DA. (**b**, **d**) miR-155-5p and (**c**) eNOS levels were determined by qRT-PCR and western blotting. (**e**) MIR155HG promoter-Luc activity was determined in cell lysates. (**f**, **g**) eNOS levels were determined by western blotting and quantitated by ImageJ. *n*=3. *P<0.05, **P<0.01 and ***P<0.001.

ROS generated by TNF-α is an important player in NF-κB activation and has also been shown to induce endothelial dysfunction by downregulating eNOS expression.^18^ We next examined the effects of CO and antioxidant on TNF-α-induced ROS generation. CORM-2 or the antioxidant *N*-acetylcysteine (NAC) inhibited intracellular ROS levels that were increased by TNF-α (Figure 8a). NAC also attenuated TNF-α-mediated miR-155-5p biogenesis, eNOS downregulation and ICAM-1 expression (Figure 8b and c). These results suggest that ROS generated by TNF-α may play an important role in miR-155-5p-mediated eNOS expression. We further examined the effect of the HO-1 byproducts on miR-155-5p biogenesis and eNOS expression in HUVECs treated with H_2_O_2_. H_2_O_2_ significantly increased miR-155-5p biogenesis and this increase was blocked by heme degradation products, except iron and NAC (Figure 8d). Similar regulatory effects of heme degradation byproducts and NAC on MIR155HG promoter activity were observed in HUVECs treated with H_2_O_2_ (Figure 8e). Furthermore, heme catabolites, except iron and NAC inhibited the H_2_O_2_-mediated eNOS downregulation and ICAM-1 upregulation (Figure 8f and g). These results suggest that HO-1 may rescue H_2_O_2_-induced eNOS downregulation by inhibiting redox-sensitive miR-155-5p biogenesis.

## Discussion

TNF-α is responsible for blood vessel malfunctions in the pathogenesis of atherosclerosis, type 2 diabetes complications and preeclampsia. The common feature that connects all of these diseases is endothelial dysfunction associated with decreased eNOS expression and activity. Endothelial function can be improved by cross-talk among endogenous gaseous molecules such as NO and CO, which are synthesized by the catalytic reaction of eNOS and HO, respectively.^25^ Multiple lines of evidence reveal that the HO-1/CO system preserves endothelial function via Akt-dependent eNOS activation^26^ and/or maintenance of eNOS expression,^17^ although the mechanism has not been clearly elucidated. Our results clearly provide new evidence that CO restores eNOS downregulation in response to TNF-α by inhibiting NF-κB-dependent biogenesis of miR-155-5p targeting the 3′-UTR of *eNOS*.

The endothelium consists of a single endothelial cell layer that lines the inner surfaces of blood vessels and plays an important role in regulating blood vessel barriers, circulation, vascular tone and inflammatory reactions. Thus, endothelial dysfunction, which is largely due to decreased eNOS expression and activity, is an important determinant of the pathogenesis of various vascular diseases, including hypertension, atherosclerosis and preeclampsia.^2, 3, 27^ Therefore, delivery of the eNOS gene and NO-donating aspirin can maintain vascular function in animal models of some vascular diseases, such as atherosclerosis and diabetic hypertension, by increasing the biological activity of the eNOS/NO pathway.^28, 29^ These findings suggest that a strategy directed at maintaining eNOS level and activity is a promising means for treating vascular diseases.

The eNOS/NO pathway is regulated by multiple mechanisms, such as decreased NO bioavailability, reduced eNOS activity and impaired eNOS expression. NO is a highly reactive free radical and is rapidly converted to inactive NO products (peroxynitrate, nitrite and nitrate) via reactions with superoxide anion or molecular oxygen, resulting in reduced NO bioactivity. The catalytic activity of eNOS is precisely tuned by the availability of the substrate L-arginine, post-translational modifications (phosphorylation at Ser^1177^ and Ca^2+^-dependent dimerization) and the endogenous NOS inhibitor asymmetric dimethyl-L-arginine.^4, 30^ In addition, eNOS expression is regulated by pathophysiological stimuli,^7, 8, 9, 11^ although it was initially considered a constitutive enzyme. In addition, estrogen transcriptionally upregulates eNOS expression. However, some inflammatory stimuli, including TNF-α, interleukin-1βLPS, downregulate eNOS expression by decreasing mRNA stability, which is blocked by NF-κB inhibition,^9^ such that eNOS has been defined as an NF-κB-responsive negative gene.^9^ In this present study, we showed that CO prevents TNF-α-mediated decreases in eNOS expression, which precludes the inflammation-induced endothelial dysfunction associated with the pathogenesis of cardiovascular diseases.

It is abundantly evident that both endogenous CO production and exogenous CO delivery demonstrate applications in a range of disease models and clinical settings.^31, 32^ We recently showed that CORM-2-preconditioned astrocytes demonstrated increases in VEGF expression and mitochondrial biogenesis via induction of HO-1, and these effects were blocked by HO-1 knockdown.^33, 34, 35^ These cellular events were synergistically increased by combined treatment with CO and bilirubin.^33, 34^ Similarly, we found that SnPP suppressed the recovery effect of CORM-2 on TNF-α-induced eNOS downregulation. Moreover, CO and bilirubin synergistically inhibited the TNF-α-mediated miR-155/eNOS pathway, suggesting that CO elicits its maximal effect by cooperating with biliverdin produced by HO-1 induction. The beneficial effects of HO-1/CO are largely linked to dual action mechanisms, such as inhibition of the NF-κB pathway and stimulation of Akt-dependent eNOS activation,^13, 26^ resulting in anti-inflammation and vasodilation in the vasculature. Consistently, our results showed that CO inhibited TNF-α-induced NF-κB activation, which is responsible for miR-155-5p biogenesis and eNOS downregulation. These effects were closely linked to a positive feedback circuit between exogenous CO and HO-1 induction, leading to the production of CO and bilirubin. Therefore, these heme catabolites generated endogenously by CORM-2-mediated HO-1 induction play an important role in maintaining eNOS expression and endothelial function.

Recent studies have demonstrated that eNOS expression is negatively regulated by a reduction of its mRNA stability via the biogenesis of miRNAs such as miR-155, miR-335, miR-543 and miR-584,^9, 36, 37^ which target the *eNOS* 3′-UTR. Among these miRNAs, miR-155-5p biogenesis is increased in HUVECs stimulated with TNF-α via transcriptional induction of the NF-κB-responsive MIR155HG, a miR-155 host gene.^9^ Our data showed that CO and bilirubin attenuated TNF-α-mediated miR-155-5p biogenesis by inhibiting the canonical NF-κB pathway, resulting in the restoration of downregulated eNOS expression. Based on the analysis of miRNA target prediction, miR-155-5p targets the *eNOS* 3′-UTR of only primates, such as humans, chimpanzees and baboons, but not other species such as mouse and rat.^18^ Thus, miR-155-5p is species-specific for the regulation of eNOS expression, which plays a crucial role in the pathogenesis of human vascular diseases.^27, 38^ However, eNOS expression in mouse and bovine endothelial cells can also be downregulated by inflammatory stimuli including TNF-α,^12, 39, 40^ suggesting that eNOS expression can be regulated in species-specific miRNAs under pathological inflammatory conditions. These findings imply that CO and bilirubin have strong therapeutic potential for cardiovascular disorders in humans rather than in other animal models, which are associated with impaired eNOS expression, likely by suppressing NF-κB-responsive miR-155-5p biogenesis.

Inappropriate activation of NF-κB is a critical step in endothelial dysfunction and downregulation of the eNOS/NO pathway.^9^ In fact, NF-κB inhibitors, such as statin and Bay11-7082, restore the attenuation of eNOS expression by TNF-α, thereby preserving endothelial function under inflammatory conditions.^9, 41^ CO and bilirubin act as anti-inflammatory molecules in endothelial cells by inhibiting NF-κB activation.^16, 42^ Although iron, another heme degradation product, has been shown to inhibit NF-κB activation, as judged by the TNF-α-driven NF-κB promoter assay following the addition of the iron chelator deferoxamine to endothelial cells overexpressing HO-1,^42^ we observed no inhibitory effect of iron on NF-κB-responsive miR-155-5p expression. This finding suggests that deferoxamine itself may inhibit the NF-κB pathway independently of the intracellular iron status. Therefore, CO and bilirubin arising from heme catabolism by HO-1 are major contributors to protection against TNF-α-induced eNOS downregulation and vascular diseases via NF-κB inhibition. A number of studies have shown that defects in HO-1 accelerate atherosclerotic lesion formation, preeclampsia and diabetic vascular dysfunction, which usually develop following abnormal elevations of TNF-α, in mouse models and human patients.^43, 44, 45^ Unfortunately, these studies did not provide evidence that the beneficial effect of HO-1/CO is linked to cross-talk between the NF-κB and eNOS/NO systems. The results of the present study demonstrated that CO and bilirubin could rescue the TNF-α-mediated eNOS downregulation and endothelial dysfunction by inhibiting NF-κB activation and miR-155-5p biogenesis.

The evidence showing that HO-1-derived CO regulates NF-κB activation provides clues to its protective effect in the context of noninfectious inflammatory vascular diseases. A number of mechanisms have been proposed to explain the inhibitory effect of HO-1 on NF-κB. For example, CO elicits anti-inflammatory effects via interference with the redox-sensitive NF-κB signaling pathway by inhibiting NADPH oxidase activation in response to LPS or TNF-α.^46, 47^ Moreover, CO inhibits vascular inflammation by interfering with the IKK-dependent signaling pathway or by stimulating *S*-glutathiolation of the NF-κB p65 subunit.^16, 47^ In addition, the HO-1-derived biliverdin/bilirubin or Fe/ferritin induction axis can inhibit TNF-α-induced reactive oxygen species production and redox-based NF-κB activation by acting as a potential antioxidant.^48, 49^ Our data showed that bilirubin, but not iron, inhibited NF-κB-responsive miR-155-5p biogenesis and eNOS downregulation in response to TNF-α, predominantly by inhibiting the canonical NF-κB activation pathway.

In addition to CO and bilirubin, biliverdin, a known antioxidant,^50^ rescued the H_2_O_2_-mediated dysregulation of the miR-155-5p/eNOS axis, although it had no protective effect in TNF-α-stimulated endothelial cells. These results suggest that CO or bilirubin could inhibit the NF-κB pathway induced by TNF-α or exogenous ROS; however, biliverdin itself may be more specific to exogenous ROS, even though it is converted to biliverdin reductase. The distinct effects of bilirubin and biliverdin may be due to their different properties, most likely the higher membrane permeability of bilirubin compared with biliverdin.^51^ Unlike biliverdin or bilirubin, CO induced HO-1 and rescued NF-κB-dependent miR-155-5p biogenesis and eNOS downregulation, which were reversed by inhibiting HO-1 activity, suggesting the presence of a positive feedback regulatory circuit between CO and HO-1 that, additionally, leads to bilirubin production. These findings provide novel evidence that CO is likely a key determinant in the homeostatic regulation of eNOS expression under inflammatory conditions.

Endothelial NO arguably plays a crucial role in vascular function; thus, decreased eNOS-derived NO production is recognized as an important risk factor in the pathogenesis of cardiovascular diseases. Our results demonstrate that CO restores TNF-α-induced eNOS downregulation and endothelial dysfunction by suppressing NF-κB-responsive miR-155-5p biogenesis. The present findings offer a mechanistic explanation for the beneficial effect of HO-1/CO on eNOS downregulation and vascular dysfunction coupled with inflammatory vascular diseases. We also provide new insight into the therapeutic potential of HO-1/CO for human inflammatory vascular diseases, including atherosclerosis, preeclampsia and diabetic vascular complications.
